# Association Mapping and Expression Analysis of the Genes Involved in the Wood Formation of Poplar

**DOI:** 10.3390/ijms241612662

**Published:** 2023-08-10

**Authors:** Yaolin Wang, Heng Zhang, Sheng Zhu, Tengfei Shen, Huixin Pan, Meng Xu

**Affiliations:** Co-Innovation Center for Sustainable Forestry in Southern China, Satae Key Laboratory of Tree Genetics and Breeding, Nanjing Forestry University, Nanjing 210037, China; wangyaolin@njfu.edu.cn (Y.W.); zhangheng19980201@163.com (H.Z.); zhusheng@njfu.edu.cn (S.Z.); tengfeishen@njfu.edu.cn (T.S.); njfuhxpan@163.com (H.P.)

**Keywords:** large-diameter timber, wood property, association analysis, transcriptional regulation, *Populus*

## Abstract

Xylogenesis is a complex and sequential biosynthetic process controlled by polygenes. Deciphering the genetic architecture of this complex quantitative trait could provide valuable information for increasing wood biomass and improving its properties. Here, we performed genomic resequencing of 64 24-year-old trees (64 hybrids of section *Aigeiros* and their parents) grown in the same field and conducted full-sib family-based association analyses of two growth and six woody traits using GEMMA as a choice of association model selection. We identified 1342 significantly associated single nucleotide polymorphisms (SNPs), 673 located in the region upstream and downstream of 565 protein-encoding genes. The transcriptional regulation network of secondary cell wall (SCW) biosynthesis was further constructed based on the published data of poplar miRNA, transcriptome, and degradome. These provided a certain scientific basis for the in-depth understanding of the mechanism of poplar timber formation and the molecular-assisted breeding in the future.

## 1. Introduction

Wood, commonly referred to as secondary xylem, produced by the vascular cambium of woody plants, is an indispensable and renewable feedstock for industrial production and daily life. Wood in the vascular cambium that programs cell death (PCD) through the maturation of cambial derivatives is considered a sequential and complex biosynthetic process driven by the coordinated expression of numerous genes involved in cell division, cell expansion, secondary cell wall (SCW) thickening, and PCD [1,2,3]. The secondary xylem of forest trees is a complex tissue comprising tracheary elements, xylem parenchyma cells, and xylem fibers. Tracheids and xylem vessels are fundamental conducting elements for the transport of water and nutrients throughout the tree body and comprise dead hollow cells with patterned SCWs. Xylem parenchyma cells are usually closely connected to tracheary elements via the remnants of plasmodesmata fields, they participate in the maintenance of xylem transport capacity, and are responsible for the recovery of vascular and tracheid function after embolism events. Xylem fibers provide mechanical support to the tree body with evenly thickened SCWs. Wood formation is characterized by massive biopolymer synthesis and deposition of biopolymers in highly thickened lignified SCWs. SCWs mainly comprise cellulose, hemicellulose, and lignin, and the content, distribution, and arrangement of these biopolymers in SCWs have important effects on wood properties.

Deciphering the genetic architecture of xylogenesis could provide valuable information for increasing wood biomass and improving its properties. With the tremendous research focused on the secondary growth of forest tree growth, the anatomical, physiological, and molecular characteristics of each stage of xylogenesis have been continuously updated from a multi-dimensional perspective [4]. A multilevel transcriptional regulatory network with NAC(NAM, ATAF1/2 and CUC2)-MYB (myeloblastosis) at its core plays a pivotal role in SCW biosynthesis [5]. With the widespread application of high-throughput sequencing technologies and genetic manipulation approaches to forest trees, our understanding of SCW biosynthesis and wood properties continues to increase [6]. However, most of this information has been obtained from saplings grown in an artificial environment. Furthermore, knowledge on the genetic architecture of adult trees grown under field conditions is still lacking. Association genetics based on high-throughput single-nucleotide polymorphism (SNP) genotyping has shown unprecedented advantages in revealing the genetic regulation mechanisms of complex quantitative traits regulated by polygenes in forest trees grown under field conditions [7,8].

Association mapping, namely linkage disequilibrium (LD) mapping, can identify allelic variations or functional genes significantly associated with complex quantitative traits by inferring the correlation between phenotypic diversity and DNA polymorphisms generated during biological evolution and then analyzing the genetic effect of alleles on phenotypes. Quantitative trait locus (QTL) mapping can be further divided into targeted candidate gene association and genome-wide association studies (GWAS). The latter has been exploited in many plants because of the dramatic reduction in genome sequencing costs. GWAS for complex traits has made significant progress not only in broad-leaved species such as poplar and eucalyptus [8], but also in coniferous species such as pine [2,9] and *Picea abies* [10]. Intriguingly, family-based GWAS designs, which could reduce bias in the estimates of direct genetic effects, have been developed and successfully applied to humans [11], chrysanthemum [12], and eucalyptus [13].

Wood properties are complex quantitative traits controlled by polygenes and vary among species and genotypes. As an ecologically and economically important hardwood species, the species of the genus *Populus* in Salicaceae, collectively known as poplars, are widely distributed in the Northern Hemisphere, from the tropics to the far northern boreal latitudes. This genus comprises five taxonomically distinct sections: *Leuce* Duby, *Tacamahaca* Spach, *Leucoides* Spach, *Aigeiros* Duby, and *Turanga* Bunge. Among them, the section *Aigeiros*, comprising *P. deltoides*, *P. nigra,* and their hybrid *P. × euramericana*, is the most important member of *Populus* for plantation culture worldwide. Both pure species and their hybrids are important commercial timber species in the mid-latitude plate of the Northern Hemisphere with fast growth, straight trunks, and broad adaptability. Poplars are an important model tree species for studying wood formation. Recently, extensive research has been conducted on secondary xylem development through association mapping and genetic manipulation experiments, and the regulatory pathways involved in poplar SCW biosynthesis have been explored.

In this study, to decipher the genetic architecture of wood traits and the correlation between wood traits and other growth traits in hybrids of *Populus* section *Aigeiros*, 66 24-year-old trees (64 hybrids and their parents) grown in the same field were resequenced and tested for wood properties. Furthermore, a full-sib family-based GWAS was conducted using the general linear model (GLM) and linear mixed model (LMM). The transcriptional regulation network of these significantly associated genes was further constructed based on the published data of poplar miRNA, transcriptome, and degradome. These results provide new insights into the improvement and cultivation of large-diameter poplar timber plantations.

## 2. Results

### 2.1. Wood Property Variation of Large-Diameter Poplars

In this study, we analyzed two growth traits (diameter at breast height (DBH) and height (H)) and six wood property traits (fiber length (FL), fiber width (FW), double wall thickness (DWT), cell lumen diameter (CLD), air-dry density (AD), and basic density (BD)) in *P. deltoides* cv. I-69 (♀), *P. × euramericana* cv. I-45 (♂), and 64 F1 hybrids (Table 1). FL, FW, DWT, AD, BD, DBH, and H of I-69 were greater than those of I-45, whereas the CLD of I-69 was lower than I-45. The greatest phenotypic values of the eight traits in the F1 hybrids were greater than those in the two parents (I-69 and I-45), indicating transgressive segregation in the full-sib family. The coefficient of variation (CV, %) of the eight traits in the F1 population ranged from 3.69 (FW) to 14.74% (DBH). Two growth traits (DBH and H) had larger CV values than the other six wood property traits. This showed there were significant differences in growth traits among these clones.

**Figure 1:**
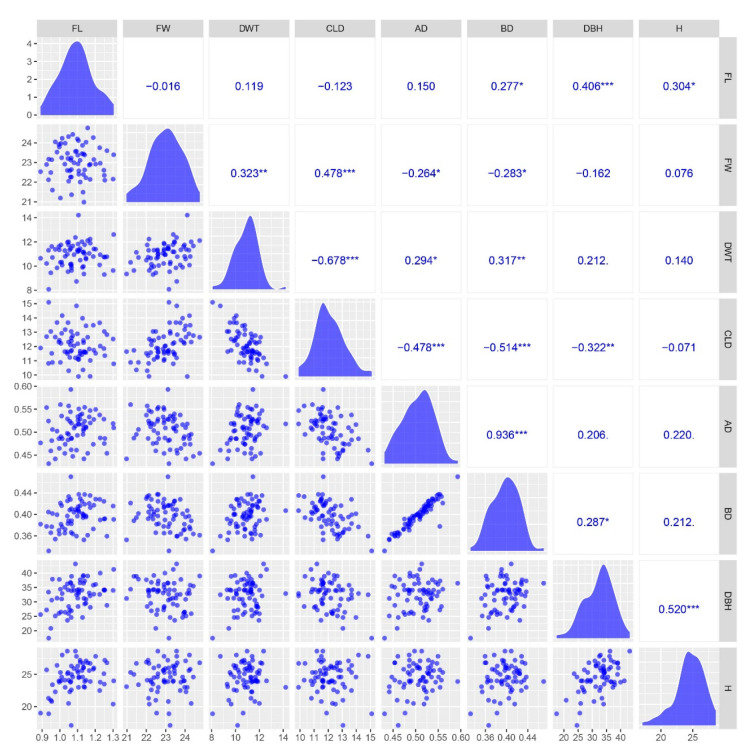
Scatterplot matrix for eight traits (fiber length (FL), fiber width (FW), double wall thickness (DWT), cell lumen diameter (CLD), air-dry density (AD), basic density (BD), diameter at breast height (DBH), and height (H)). Lower triangle: Scatter plots of units between pairs of eight poplar traits. Diagonal: density plot of eight traits. Upper triangle: correlation coefficient (Pearson’s r) between the eight traits within the full-sib poplar family. *, 0.01 < *p* < 0.05; **, 0.001 < *p* < 0.01; ***, *p* < 0.001.

The distribution of the eight traits exhibited an approximate bell shape (Figure 1). Using the Shapiro–Wilk test for the eight phenotypic datasets, the W statistic was close to 1 and *p* > 0.05 (Table S1), indicating that the datasets conformed to a normal distribution and were suitable for further GWAS analysis. The pairwise correlation analysis indicated there was no strong linear relationship among the eight traits, except the pair of AD and BD (Figure 1). The correlation between AD and BD was strong (r^2^ = 0.94), showing a significant correlation level.

### 2.2. Genome-Wide Variations in a Full-Sib Population

All approximately 671.6 Gbp raw reads (3,060,185,242) were generated through the Illumina whole-genome resequencing of all 66 clones. After filtering, 203,819,202 (51.5×) and 210,409,496 (53.4×) clean reads were obtained from parents I-69 and I-45, respectively. A total of 2,608,389,006 clean reads were generated for 64 F1 hybrids, with a mean sequencing depth of 9.9× each hybrid, ranging from 6.0 to 13.8 (Table S2). We identified 183,253 insertion variants, 214,337 deletion variants, and 7,800,813 SNPs from the clean sequencing data of 66 poplars (Table S3). A total of 397,590 InDels (insertions and deletions) were unevenly distributed across 19 poplar chromosomes, with an average density ranging from 749 (Chr19) to 1271 (Chr09) per Mbp (Figure S1). This also happens with SNPs and relatively more SNPs were located in the central region of all the chromosomes, except Chr08, Chr09, and Chr14 (Figure S2).

**Figure 2:**
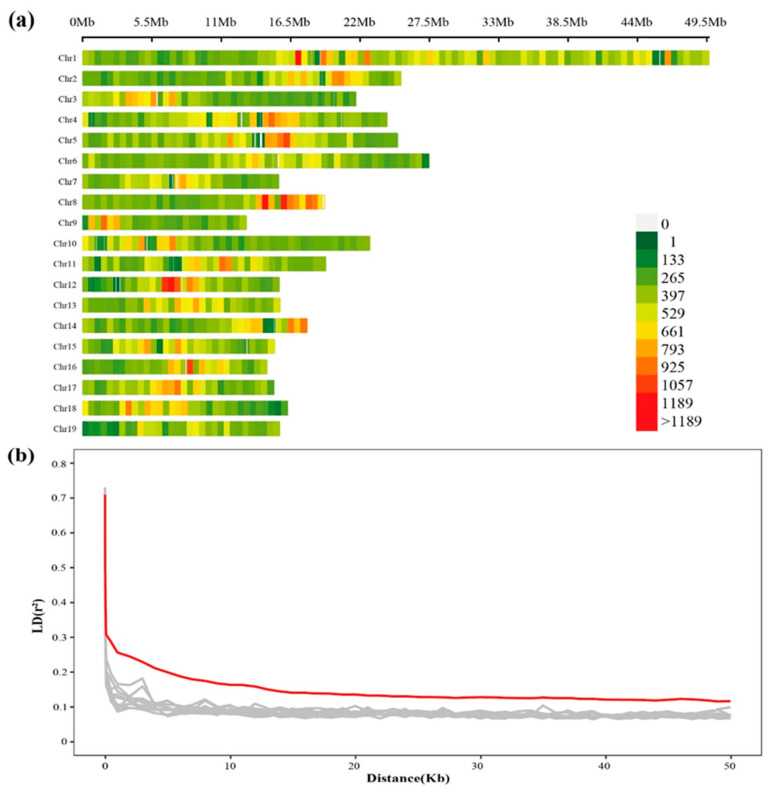
(**a**) SNP (single-nucleotide polymorphism) density plot across all 19 poplar chromosomes. The horizontal axis at the top represents the physical location (Mbp) on the chromosome. The color bar indicates the number of SNPs within the size of the 0.5 Mbp window. (**b**) Plot of the LD (linkage disequilibrium) rate (r^2^) against the physical distance in Kbp. The red line indicates the pattern of LD decay for the entire genome, and the gray line represents the LD decay for each of 10 randomly selected chromosomes.

In total, 332,639 high-quality SNPs were selected under stringent quality control metrics for subsequent LD and GWAS analyses. The mean number of high-quality SNPs was 855 per Mbp window, ranging from 731 per Mbp on Chr19 to 963 per Mbp on Chr08 (Figure 2a). All selected SNPs were divided into six types of nucleotide substitution: A to G (G to A), C to T (T to C), A to T (T to A), A to C (C to A), G to T (T to G), and G to C (C to G). The ratio of transition (Ti) to transversion (Tv) is an indicator used to evaluate the quality of SNPs in a genome [14,15,16,17]. The Ti/Tv ratio of poplar SNPs was 2.44 (Figure S3), which is similar to that of rice [18], chicken [19], and humans [20].

### 2.3. Genetic Recombination and LD Decay

LD was estimated by calculating the squared correlation coefficient (r^2^) between intrachromosomal SNP pairs within the full-sib population. We plotted LD decay along the entire poplar genome using 332,639 high-quality SNVs in 66 poplar clones from a full-sib family. LD decay is an index of genetic recombination in the biparental population [21]. The LD decay distance can estimate the minimum number of molecular markers required for subsequent GWAS [22]. Randomly selected chromosomes have approximately the same LD decay pattern between them (Figure 2b). The average physical distance at which the LD value (r^2^) for the whole genome decayed below 0.2 was approximately 3 Kbp, which was like the LD decay distance of 2.6 Kbp (r^2^ = 0.2) in *P. euphratica* [23]. This indicates that at least 133,000 SNPs are necessary for further GWAS [24]. Thus, it was likely sufficient to perform a GWAS on a dataset of 332,639 high-quality SNPs.

### 2.4. Association Model Selection

**Figure 3:**
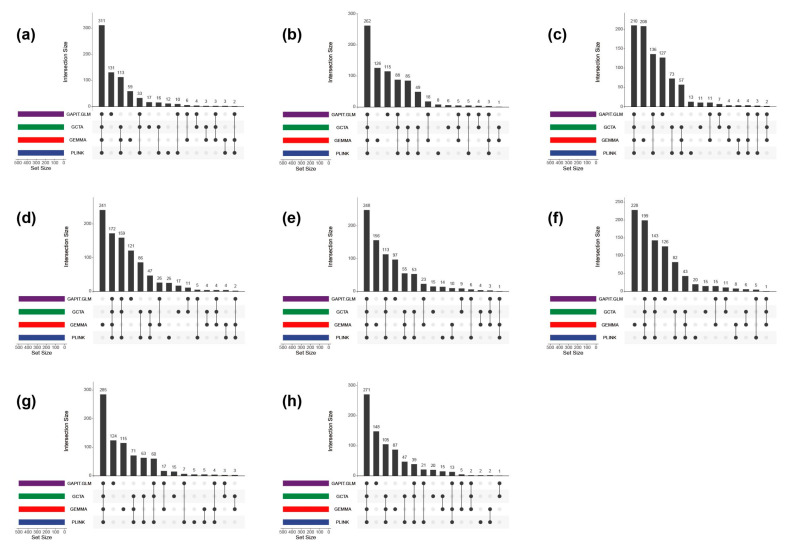
Upset plot of the top 500 SNPs (single-nucleotide polymorphisms) of four methods GWAS (genome-wide association studies) results for eight poplar traits. Subplot (**a**): fiber length (FL). Subplot (**b**): fiber width (FW). Subplot (**c**): double wall thickness (DWT). Subplot (**d**): cell lumen diameter (CLD). Subplot (**e**): air-dry density (AD). Subplot (**f**): basic density (BD). Subplot (**g**): diameter at breast height (DBH). Subplot (**h**): height (H). Each row in the lower panel represents a method, and the corresponding colored bars on the lower left represent the number of each method, whereas the main bar plot (top) is the number of SNPs in different sets.

Two classical GWAS models, LMM (e.g., GEMMA and GCTA) and GLM (e.g., GAPIT and PLINK), have been applied to map QTLs associated with complex traits in plants [25]. In this study, we used four tools, GEMMA, GCTA, GAPIT, and PLINK, to associate eight traits with 332,639 high-quality SNPs from a full-sib family. To compare the association analysis results using all four approaches, we selected the top 500 trait-associated SNPs based on the *p*-value. A total of 723 SNPs were associated with FL, 775 SNPs for FW, 870 SNPs for DWT, 924 SNPs for CLD, 807 SNPs for AD, 902 SNPs for BD, 777 SNPs for DBH, and 778 SNPs for H. The number of SNPs identified using all four methods ranged from 172 (CLD) to 311 (FL) (Figure 3). The numbers of SNPs identified by a single tool were 219 SNPs for FL, 255 SNPs for FW, 359 SNPs for DWT, 404 SNPs for CLD, 282 SNPs for AD, BD, 259 SNPs for DBH, and 257 SNPs for H. The similarity between the association results obtained using the four tools was the difference between the eight trait datasets (Figure 3).

**Figure 4:**
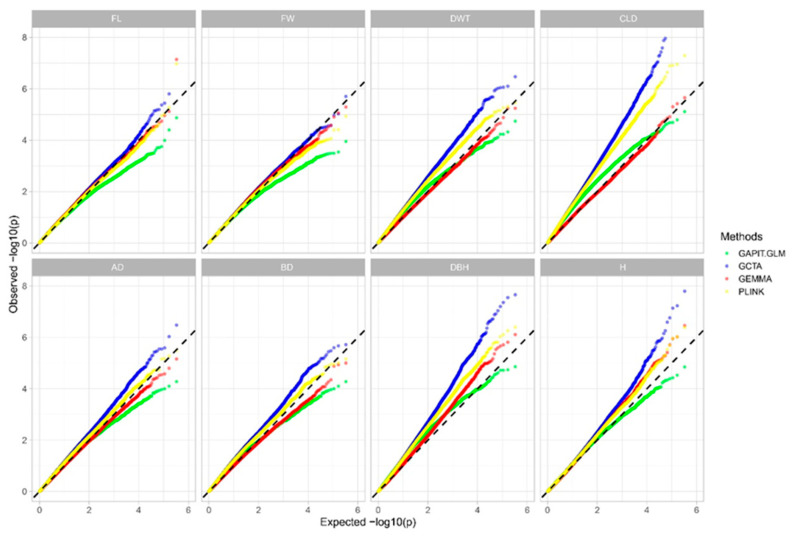
Quantile–quantile plot (QQ plot) of fiber length (FL), fiber width (FW), double wall thickness (DWT), cell lumen diameter (CLD), air-dry density (AD), basic density (BD), diameter at breast height (DBH), and height (H) for four GWAS (genome-wide association studies) methods (GAPIT.GLM, GCTA, GEMMA, and PLINK).

The QQ (Q-Q) plot and the genomic inflation factor (λ statistic) are two frequently used indicators for evaluating GWAS models/tools [26,27]. No one method is significantly better than the others for all eight traits (Figure 4). However, similar results were observed in the genomic inflation factor (λ statistic) analysis. GEMMA obtained a λ value closer to one in six traits (e.g., DWT, CLD, AD, BD, DBH, and H) than the other three association tools (Table 2). Although the best λ values for the remaining two traits (FL and FW) were obtained from the GAPIT.GLM results, the top two λ values were close to those of the GEMMA results. The average λ (1.04) value of the eight traits using GEMMA was closer to 1 than those of the other three association methods (Table 2). Therefore, we selected GEMMA as the GWAS analysis tool, and the GEMMA association results were used for further analysis.

### 2.5. Full-Sib Population-Based Association Mapping

**Figure 5:**
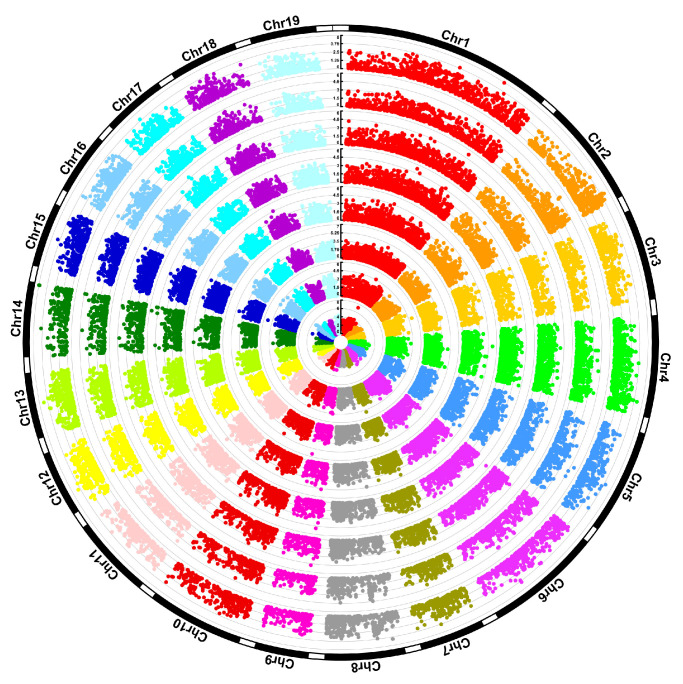
Manhattan plots of the GWAS (genome-wide association studies) results for eight poplar characters. SNPs (single-nucleotide polymorphisms) were removed when *p* > 0.005 in all traits from inside to outside. Fiber length (FL), fiber width (FW), double wall thickness (DWT), cell lumen diameter (CLD), air-dry density (AD), basic density (BD), diameter at breast height (DBH), and height (H). Notes: when *p* > 0.0005.

In this study, we identified 1342 significantly associated SNPs for all eight traits using GEMMA below the threshold of *p* < 0.0005 (Figure 5). In total, 376 and 370 SNPs were significantly associated with DBH and H, respectively. The number of two growth-trait-related SNPs was greater than the six wood traits (117 for FL, 138 for FW, 116 for DWT, 121 for CLD, 120 for AD, and 69 for BD). Approximately half (673) of the 1342 significant SNPs were related to the 565 protein-encoding genes in the *P. trichocarpa* (v4.1) genome. There were more genes associated with growth traits (177 genes for DBH and 129 genes for H) than with the six wood traits (58 genes for FL, 48 genes for FW, 50 genes for DWT, 54 genes for CLD, 62 genes for AD, and 31 genes for BD) (Table S4).

In total, 342 of the 565 significant genes were assigned functional information in the functional annotation file of the *P. trichocarpa* (v4.1) genome. Many genes belong to the same gene family, including four *R2R3-type* MYB and three *LAC* (laccase) family genes. A total of 41 genes were significantly associated with two traits (AD and BD, DBH and H, and DWT and CLD), and only one gene was significantly associated with three traits (CLD, AD, and BD) (Table S5). There were 18 genes associated with AD and BD traits, possibly due to the high correlation coefficient (r^2^ = 0.94). A total of 12 genes associated with DBH and H were identified, due in part to the high similarity in the morphogenesis of DBH and H. The GO and KEGG annotations of the 565 significant genes are listed in Tables S6 and S7. In the GO enrichment analysis, for wood traits, enrichment was in hydrolase activity, acting on ester bonds, ribosome, nucleosome, structural constituent of ribosome, and translation pathway, and for growth traits were enriched in peroxisome, cis-Golgi network, endoplasmic reticulum to Golgi vesicle-mediated transport, cytochrome-c oxidase activity, and ADP binding pathway (Table S6). In the KEGG enrichment analysis, wood traits were mainly enriched in ribosome, endocytosis, isoquinoline alkaloid biosynthesis, lysine degradation, sesquiterpenoid, and the triterpenoid biosynthesis pathway, while for growth traits, the main enrichments were in cysteine and methionine metabolism, glyoxylate and dicarboxylate metabolism, and the endocytosis pathway (Table S7).

### 2.6. Transcriptional Regulatory Network for SCW Biosynthesis

We adopted fragments per kilobase of transcript per million mapped reads (FPKM) values, which were used to measure the expression levels of these candidate genes in two poplar clones: CL290 (clone 290 with fast growing) and CL33 (clone 33 with slow growing). Of the 565 candidate genes, 143 (25.31%) and 165 (29.20%) were highly expressed (Expression > 10) in CL290 and CL33 cells, respectively (Figure S3, Table S8). We identified 38 differentially expressed genes (DEGs) (log2 FoldChange > 2) between CL290 and CL33 cells. A total of 21 and 17 genes were upregulated in CL290 and CL33 cells, respectively (Table S9).

**Figure 6:**
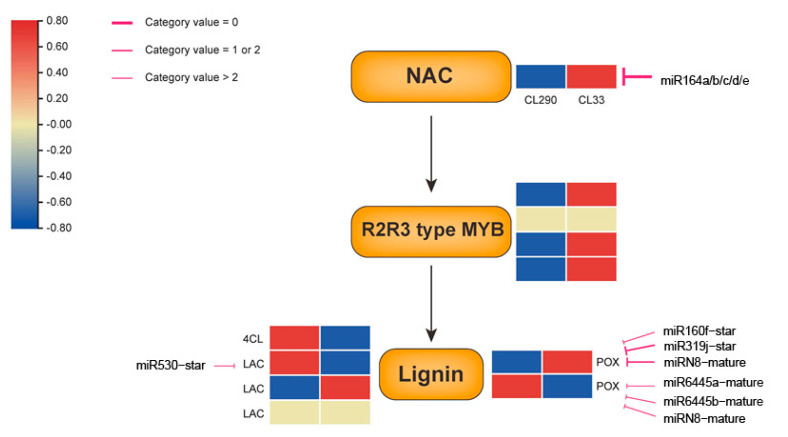
Typical pathways of the NAC-containing gene. The “T” arrows indicate interacting miRNAs, and the shades of color represent different category values.

A total of 119 candidate genes were regulated by 128 miRNAs (Figure S4), which comprised 270 gene-miRNA regulatory pairs (Table S10). There were 51 candidate genes targeted by more than one miRNA. For example, the gene Potri.003G050100, which encodes the HD-ZIP protein, is regulated by 14 members of the miR166 family, suggesting that the miR166-HD-ZIP module could play an important role in poplar wood formation. Potri.006G054100, which belongs to the superfamily of ARM repeat proteins, is targeted by six members of the miR167 family. The NAC-containing gene Potri.012G001400 interacted with five members of the miR164 family (Figure 6).

## 3. Discussion

Population structure is a frequent consideration in GWAS, and the greater the differences between populations, the easier it is for GWAS to identify them. Therefore, most contemporary studies usually aim to sample natural populations with rich genetic diversity, which is more suitable for GWAS [28]. However, it was found that growth variation in the offspring of artificial interspecific crosses was significantly genetically different, showing a great deal of variability [29]. Therefore, artificial populations of half-sib or full-sib families with a clear genetic background and rich variation have become widely studied in forest tree breeding [30,31,32]. In addition, although the genomic recombination rate in the artificial breeding population is low, it can amplify the frequency of rare mutation sites and detect some additional significant associations through adjusting the threshold of the *p*-value [32], also a more convenient and simpler method of preserving germplasm resources than natural populations [33]. Therefore, there is potential and feasibility to utilize a full-sib artificial hybrid population of *P. deltoides* with *P. × euramericana* for GWAS in this study.

LD is related to genetic recombination and is used to estimate the number of markers required for GWAS [34]. The resolution and number of markers required for GWAS depend on the extent of LD [35]. The LD decay was different among species in the genus *Populus*, an LD decay ranged from 1.4 to 5.1 Kbp (r^2^ threshold of 0.2) was reported in *P. deltoides* [36], and an LD decay of 2.6 Kbp was reported in *P. euphratica* at r^2^ = 0.2 [23]. In this study, the LD value (r^2^) for the whole genome decayed below 0.2 was approximately 3 kb, indicating that at least 134,000 markers were required [37]. This shows that 332,639 SNPs met the number of markers required for full-sib family-based GWAS. The QQ plot and genomic inflation λ are two metrics commonly used to select an optimization association model [26,27]. Yang et al. selected a model to associate with the leaves of the F1 pedigree of *P. deltoides* and *P. simonii* based on good genomic control (genomic inflation equal to 1) [32]. Furthermore, Zhang et al. selected the most effective model for poplar GWAS based on the degree of deviation between the observed and expected *p values* [34]. However, the QQ plot and λ metric suggested that no one association tool could surpass the performance of the other three tools for the eight traits, due to the possible absence of population stratification and the presence of full-sib kinship in the I-69 and I-45 families [38]. We selected GEMMA as the final GWAS model based on the average λ metric for all eight traits. GEMMA is a GWAS toolkit to rapidly implement an LMM, with population structure as a fixed effect and kinship as a random effect [39,40].

Wood formation is a complex process in which transcription factors play important regulatory roles in various processes of xylem development, including transcription factor families such as HD-ZIP, MYB, and NAC. The MYB family, as one of the largest families of transcription factors in plants, not only plays a role in plant stress, but also plays an important regulatory role in xylem development, and several MYB transcription factors related to wood formation were associated in this study. Among them, MYB118 has been verified in *Arabidopsis thaliana* to have the ability to induce plant-to-embryo transition, the formation of somatic embryos from root explants [41], and to play a regulatory role in xylem conduit development [42]. MYB59 is identified in *A. thaliana* to be involved in the transcriptional regulation of secondary xylem formation [43]. Several transcription factors have also been identified in poplar associated with secondary wall development, such as Poplar PtrMYB2, PtrMYB3, PtrMYB20, and PtrMYB21 [44]; (Homologs of MYB46 and MYB83 in *A. thaliana*), PtrMYB55, and PtrMYB121 [45]; (Homologs of MYB55 in *A. thaliana*), PtoMYB92, and PtoMYB125 [46]; (Homologs of MYB85 in *A. thaliana*), PtoMYB170 [47], and PtoMYB216 [48]; and (Homologs of MYB61 in *A. thaliana*) were identified to be involved in regulating secondary cell wall formation. Conversely, PtrMYB6 [49], PtoMYB156 [50], PtrMYB189 [51], and PdMYB221 [52] had inhibitory effects on lignin accumulation. Previous studies have shown that, like *MYB* genes, NAC transcription factors play an important regulatory role in xylem development [53]. The NAC080 associated in this study was identified as a transcription factor related to lignin biosynthesis in *Brassica napus* [54]. In poplar, PtrWND 1A, PtrWND 1B, PtrWND 2A, and PtrWND 2B [55] (Homologs of NST 1, NST 2, and SND 1 in *A. thaliana*), were identified to affect secondary wall thickness. *Peroxidase* (*Pox*), *laccase* (*LAC*), and *4-coumarate-CoA ligase* (*4CL*) are key genes involved in lignin synthesis. The laccase gene *LAC17*, identified in this study, was characterized in *A. thaliana* for the lignification of vascular cells [56]. The *4CL1* gene has been identified as being involved in lignin biosynthesis based on molecular and reverse genetic characterization in *P. tremula × P. alba* ‘INRA-France 717-1B4′ [57]. MiRNAs that regulate lignin biosynthesis have been reported in many plants, including maize [58], *Arabidopsis* [59], and poplar [60]. In this study, one *NAC* gene, one *LAC* gene, and two *Pox* protein-encoding genes were regulated by miRNAs. These gene-miRNA regulatory pairs may play important roles in wood formation.

In this study, we performed resequencing using GWAS on 64 full-sibling families derived from a cross between *P. deltoides* cv. I-69 and *P. × euramericana* cv. I-45. We determined six wood traits (FL, FW, DWT, CLD, AD, and BD) and two growth traits (H and DBH), and identified several SNP loci associated with these material traits. Additionally, we constructed a transcriptional regulatory network of SCW biosynthesis through transcriptome analysis. These findings provide a solid scientific foundation for gaining a deeper understanding of the mechanism behind poplar timber formation and for future molecular-assisted breeding efforts.

## 4. Materials and Methods

### 4.1. Plant Materials

In the 1970s, excellent clones of section *Aigeiros* from Europe and the United States were introduced into China. They have had strong adaptability and rapid growth in the plains of Huanghuai and Jianghuai, and in the middle and lower reaches of the Yangtze River [61]. Since then, researchers have conducted a series of artificial hybridizations of the section *Aigeiros*. The full-sib population used in this study was derived from a cross between *P. deltoides* cv. I-69 and *P. × euramericana* cv. I-45 in the early 1980s. The full-sib progenies, along with I-69 and I-45, were planted and spaced in a 6 × 6 m configuration at the Zhangji Forest Farm (34.14° N, 117.38° W) in Jiangsu Province, China. In total, 66 24-year-old trees (64 progenies and 2 parents) were selected for genome resequencing and wood property measurement.

### 4.2. Wood Property Determination

For each clone, discs (50 mm thick) were collected at ground level and at 1.3 m. The wood was sampled in two ways, the first was to take wedge-shaped strips of the discs cut radially through the center of the circle, and then split the wedge-shaped blocks into matchstick size for fiber morphology determination. The second was to take 2 cm × 2 cm strips from the center of the circle, and then quintupling the samples into five pieces of wood, including early wood and late wood, for the determination of BD and AD.

To measure fiber morphology using the Franklin method [62], the wood samples were put into a beaker and repeatedly boiled in water until the wood strips sank. The water was discarded and glacial acetic acid was added to the beaker in the volume ratio of 1:1. Hydrogen peroxide was mixed into the solution for dissociation and placed at room temperature for one week. Once the specimens became white and fluffy, they were removed and the acid was discarded. The specimens were washed with deionized water 4–5 times until the acid was washed out and the specimens were neutral. The specimens were broken into single fibers with a disperser, and then the instrument Optest FQAII (OpTest Equipment Inc., Hawkesbury, ON, Canada) was used to set up 2000 fibers, and FL, FW, DWT, and CLD were determined according to the operation procedure. The average of 3 groups was taken for each sample.

BD was determined by soaking the wood blocks in deionized water, changing the water every 2 days to prevent rotting and deterioration, for 2 weeks until the samples were saturated with water. The samples were taken out and the volume of the wood blocks was read on a GP-120W Wood Basic Density Tester (MatsuHaku Inc., Taiwan, China) (accurate to 0.001 cm^3^). The samples were then placed in a BINDER/ED Series oven (Binder Inc., Tuttlingen, Germany) and dried at 105 °C until constant weight (about 48 h), and then quickly weighed with an L-IC precision electronic balance (METTLER TOLEDO Inc., Greifensee, Switzerland) (accurate to 0.0001 g) after drying and cooling. BD was calculated according to the following formula: ρ = m_0_/V_max_ where ρ is the basic density of the wood block (g/cm^3^); m_0_ is the weight of the wood block at full dryness (g); and V_max_ is the saturated volume of the wood block (cm^3^).

AD was determined by placing the wood blocks at room temperature to constant weight (27 °C, 1–2 months) and measuring AD using the sealing wax method on a GP-120W Wood Basic Density Tester (accurate to 0.001 g).

### 4.3. Genome Resequencing and SNP Genotyping

Genomic DNA was extracted from young leaves using the DNeasy Plant Mini Kit (Qiagen, Hilden, Germany). DNA libraries were constructed and sequenced on an Illumina HiSeq2000 platform to generate raw paired-end (PE) reads. Raw reads were trimmed using Fastp (v.0.23.2) [63] with the following parameter settings: (a) reads with ≤5 unidentified nucleotides (N), (b) trimmed reads with a minimum length of 60 bp, and (c) reads with >40% bases having a minimal Phred-scaled quality of 15. The trimmed reads were mapped against the *P. trichocarpa* (v4.1, https://phytozome.jgi.doe.gov accessed on 16 July 2022) genome using bwa-mem2 (v.2.0pre2, https://github.com/bwa-mem2/bwa-mem2 accessed on 16 July 2022). Sequence alignment format (SAM) files were converted to binary SAM (BAM) files using SAMtools (v.1.9) [64]. PCR duplicates were eliminated using MarkDuplicates implemented in the Picard software package (v.2.27.4; https://github.com/broadinstitute/picard/releases/ accessed on 16 July 2022). Raw DNA variants were called using mpileup implemented in BCF tools (v.1.9; http://samtools.github.io/bcftools/ accessed on 16 July 2022). Tri- and tetra-allelic SNPs were discarded from the raw variants. The identified variants were filtered using BCFtools under the following parameters: (a) SNPs within 5 bp of an InDel, (b) SNPs with a missing rate of >5%, (c) SNP with a <5% minor allele frequency (MAF), (d) SNPs detected at an average depth of <5×, and (e) Hardy–Weinberg equilibrium (HWE) test *p* > 10^−6^ using PLINK (v.1.9, https://www.cog-genomics.org/plink/ accessed on 17 July 2022) [65].

### 4.4. LD Decay Analysis

These high-quality SNPs in the VCF format were used for the LD decay analysis using PopLDdecay (v.3.42) with default parameters [66]. The average r^2^ value between the SNP markers was assessed for each of the 19 chromosomes and the entire genome within the full-sib population. The LD decay was plotted using the distance against the average r^2^ value within 50-Kbp windows the R package ggplot2 (v.3.3.6).

### 4.5. Statistical Algorithms

Four software programs—GLM of GAPIT (v.3.0) [67], GLM of PLINK [65], LMM of GEMMA (v.0.98.5) [68], and LMM of GCTA (v.1.94.1) [69]—were used for the LD analysis. *p* < 0.0005 was considered significant. Manhattan and quantile–quantile plots (QQ plot) were visualized using the R package CMplot (v.4.1.0, https://github.com/YinLiLin/CMplot accessed on 5 August 2022).

The top 500 SNPs were selected from the eight traits to compare the models. The intersection of the top SNPs was visualized using the UpSetR R package (v.1.4.0) [70]. The most appropriate method for our full-sib family was selected using the average genomic inflation factor (λ statistic). The genomic inflation factor (λ statistic) had a wide range of values for different methods, suggesting that different methods can affect the accuracy of the GWAS. The λ statistic value was calculated based on the result of the GWAS analysis by determining a ratio between the median of the resulting chi-squared test statistics and the expected median of the chi-squared distribution in by R package GWAS.utils (v.0.1, https://github.com/sinarueeger/GWAS.utils accessed on 5 August 2022) [71,72].

### 4.6. Enrichment and Transcriptional Expression Analysis

The potential function annotation, Gene Ontology (GO), and Kyoto Encyclopedia of Genes and Genomes (KEGG) information were obtained from the *P. trichocarpa* v4.1 assembly and annotation. Enrichment analyses were performed using the ClusterProfiler R package (v4.2.2; https://github.com/YuLab-SMU/clusterProfiler accessed on 5 August 2022) [72]. Gene network analysis was performed on candidate genes using STRING (v.11.5, https://cn.string-db.org/ accessed on 5 August 2022). Transcriptome, sRNA, and degradome data were obtained from a previous study [73].

## 5. Conclusions

In this study, we conducted an association study of two growth traits and six woody traits of 66 24-year-old clones from an *Aigioros* full-sib family, using high-quality 332,639 SNPs exceeding the LD-based estimate of markers required for GWAS. We identified 1342 SNPs and 565 protein-encoding genes significantly associated with the eight poplar traits. Published poplar multiomics data (e.g., miRNA, transcriptome, and degradome) have demonstrated that some of the significantly associated genes, such as *NAC*, *R2R3-type MYB*, and lignin-related genes, could be involved in wood formation. These results elucidate information for dissecting the molecular mechanism of wood formation and developing new poplar varieties with large-diameter timber.

## Figures and Tables

**Table 1:** Phenotypic information (fiber length (FL), fiber width (FW), double wall thickness (DWT), cell lumen diameter (CLD), air-dry density (AD), basic density (BD), diameter at breast height (DBH), and height (H)) of *P. deltoides* cv. I-69 and *P. × euramericana* cv. I-45 and their F1 Population.

Traits	Male	Female	F1 Population			
			Mean ± SD	Min	Max	CV (%)
FL (mm)	0.94	1.14	1.09 ± 0.09	0.89	1.30	8.55
FW (μm)	23.17	23.38	23.00 ± 0.85	20.98	24.75	3.69
DWT (μm)	8.07	11.77	10.93 ± 0.95	8.72	14.21	8.65
CLD (μm)	15.09	11.61	12.07 ± 1.03	9.90	14.84	8.51
AD (g/cm^3^)	0.43	0.52	0.51 ± 0.03	0.44	0.59	6.59
BD (g/cm^3^)	0.33	0.42	0.40 ± 0.2	0.35	0.47	6.23
DBH (cm)	17.40	29.30	32.61 ± 4.81	20.80	43.20	14.74
H (m)	18.90	23.90	24.59 ± 2.28	17.10	28.60	9.89

**Table 2:** Genomic inflation factor (λ statistic) of the GWAS (genome-wide association studies) results of eight traits (fiber length (FL), fiber width (FW), double wall thickness (DWT), cell lumen diameter (CLD), air-dry density (AD), basic density (BD), diameter at breast height (DBH), and height (H)) using four tools.

	GAPIT.GLM	GCTA	GEMMA	PLINK
FL	1.116	1.144	1.153	1.131
FW	1.060	1.093	1.062	1.080
DWT	1.484	1.507	1.024	1.504
CLD	1.737	1.855	1.000	1.866
AD	1.177	1.201	1.009	1.187
BD	1.181	1.422	1.047	1.407
DBH	1.498	1.379	1.028	1.376
H	1.101	1.068	0.981	1.071
Average λ	1.301	1.340	1.040	1.334

## Data Availability

Sequencing data generated in the study are available in the NCBI Sequence Read Archive (SRA) under BioProject accession PRJNA1001796.

## Supplementary Materials

The supporting information can be downloaded at: https://www.mdpi.com/article/10.3390/ijms241612662/s1.
