# Supplementary material for: Association Mapping and Expression Analysis of the Genes Involved in the Wood Formation of Poplar
Source: Int J Mol Sci. 2023 Aug 10;24(16):12662. doi: 10.3390/ijms241612662 (PMC10454019; doi:10.3390/ijms241612662)
Supplement: Supplementary file 1 [file ijms-24-12662-s001.zip › captions.pdf]

Table S1. Shapiro-Wilks test in 8 wood property traits.
Table S2. Populus clones re-sequencing raw reads, clean reads, mapping reads, raw depth, clean depth, and mapping depth.
Table S3. The statistics and average (Mbp) densities of SNP and InDel on each chromosome.
Table S4. Corresponding gene of Significant SNP in eight poplar traits, with the significance threshold level of $p < 0.0005$.
Table S5. Genome-wide significant candidate genes associated with more than one trait.
Table S6. Information on the GO annotated from the corresponding gene with eight poplar traits.
Table S7. Information on the KEGG annotated from the Corresponding gene with 8 wood property traits.
Table S8. Expression level of the candidate genes in CL290 and CL33.
Table S9. Differential expression of candidate genes between CL290 and CL33.
Table S10. miRNA related to the candidate genes.
Figure S1. The number of indels within 1Mb window size.
Figure S2. The number of SNPs within 1Mb window size.
Figure S3. Alternative splicing sites.
Figure S4. Expression of CL290 and CL33.
