# Supplementary figures and images for: Association Mapping and Expression Analysis of the Genes Involved in the Wood Formation of Poplar
Source: Int J Mol Sci. 2023 Aug 10;24(16):12662. doi: 10.3390/ijms241612662 (PMC10454019; doi:10.3390/ijms241612662)

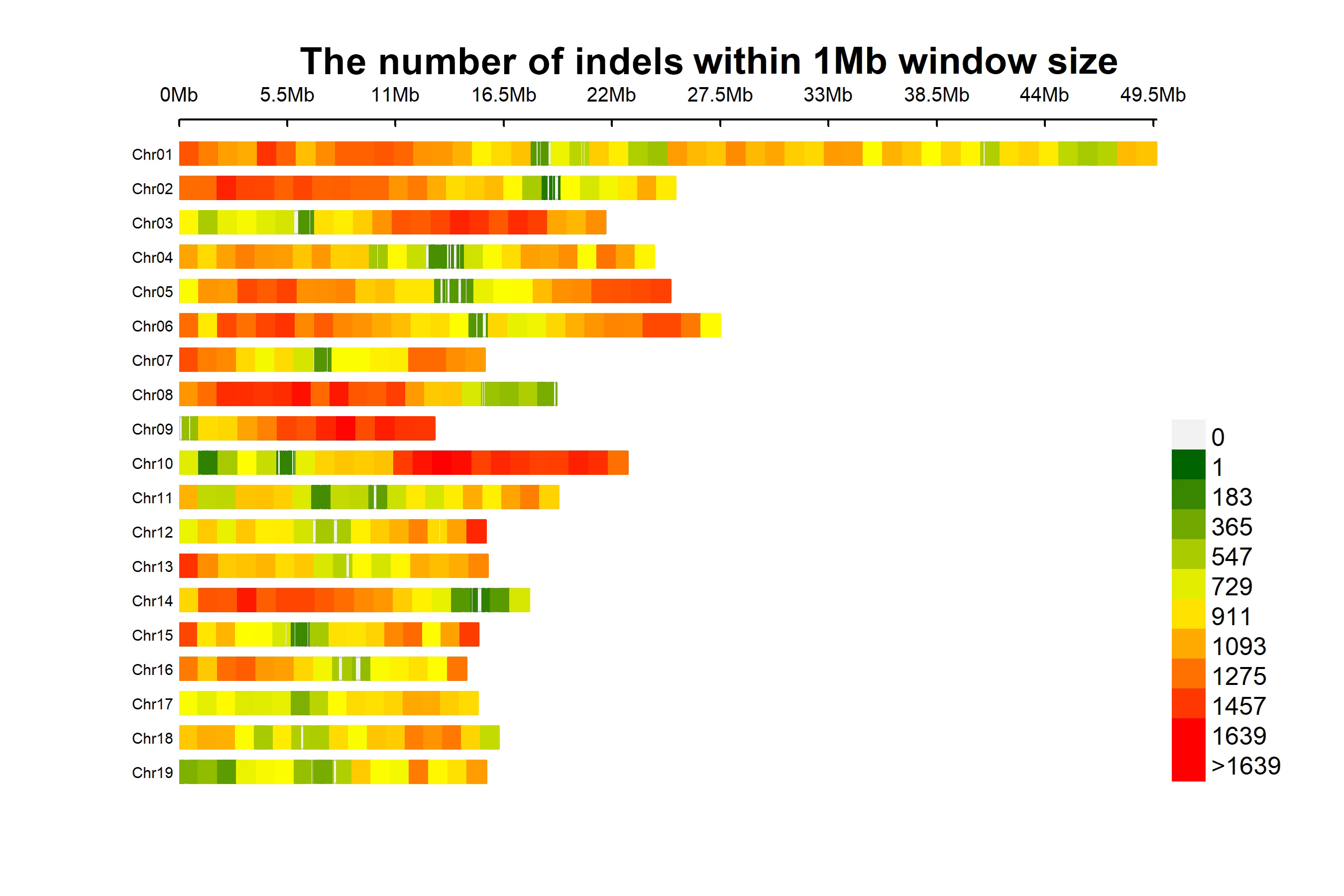

Supplement: Supplementary file 1 [file ijms-24-12662-s001.zip › Figure S1. The number of indels within 1Mb window size.jpg]

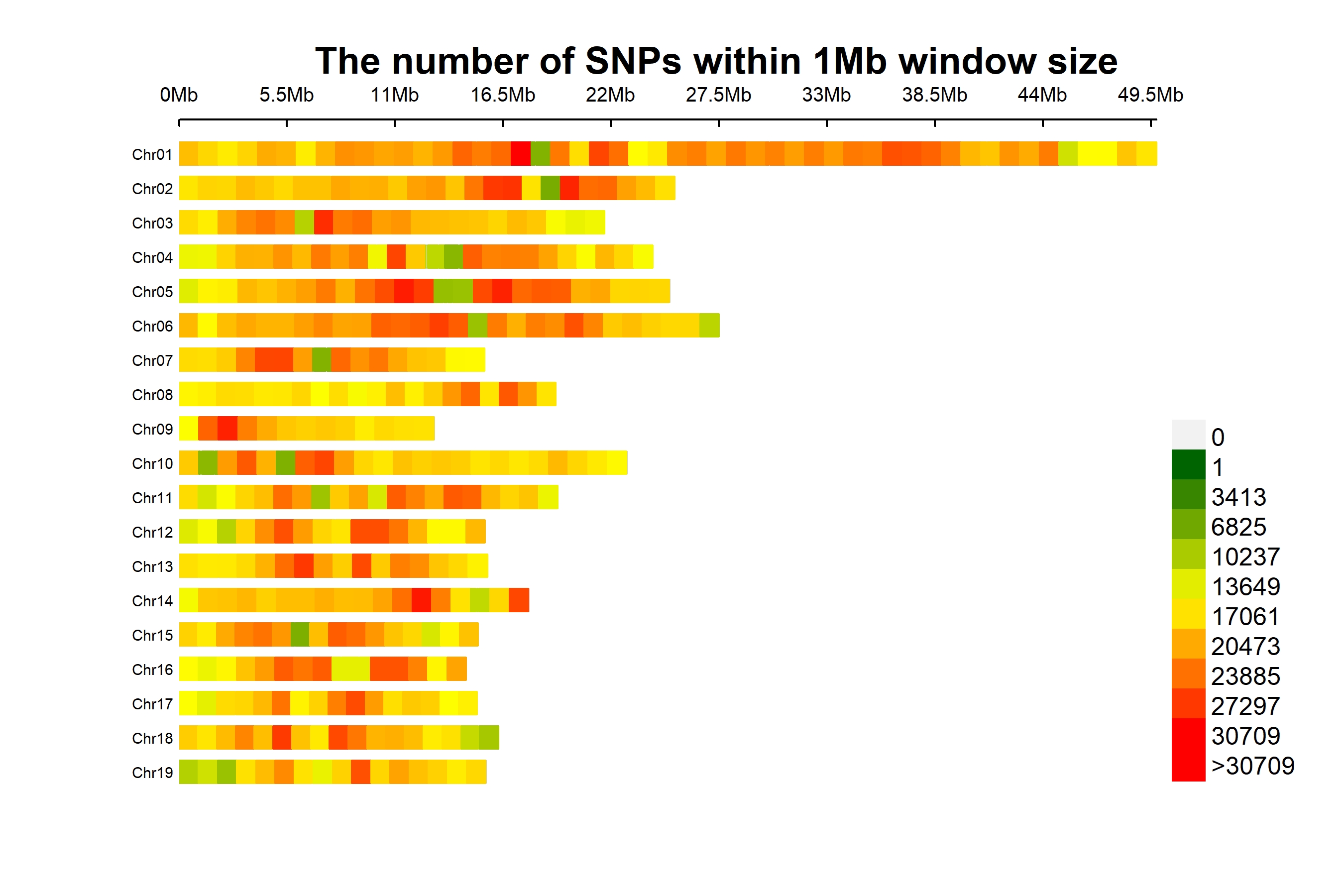

Supplement: Supplementary file 1 [file ijms-24-12662-s001.zip › Figure S2. The number of SNPs within 1Mb window size.jpg]

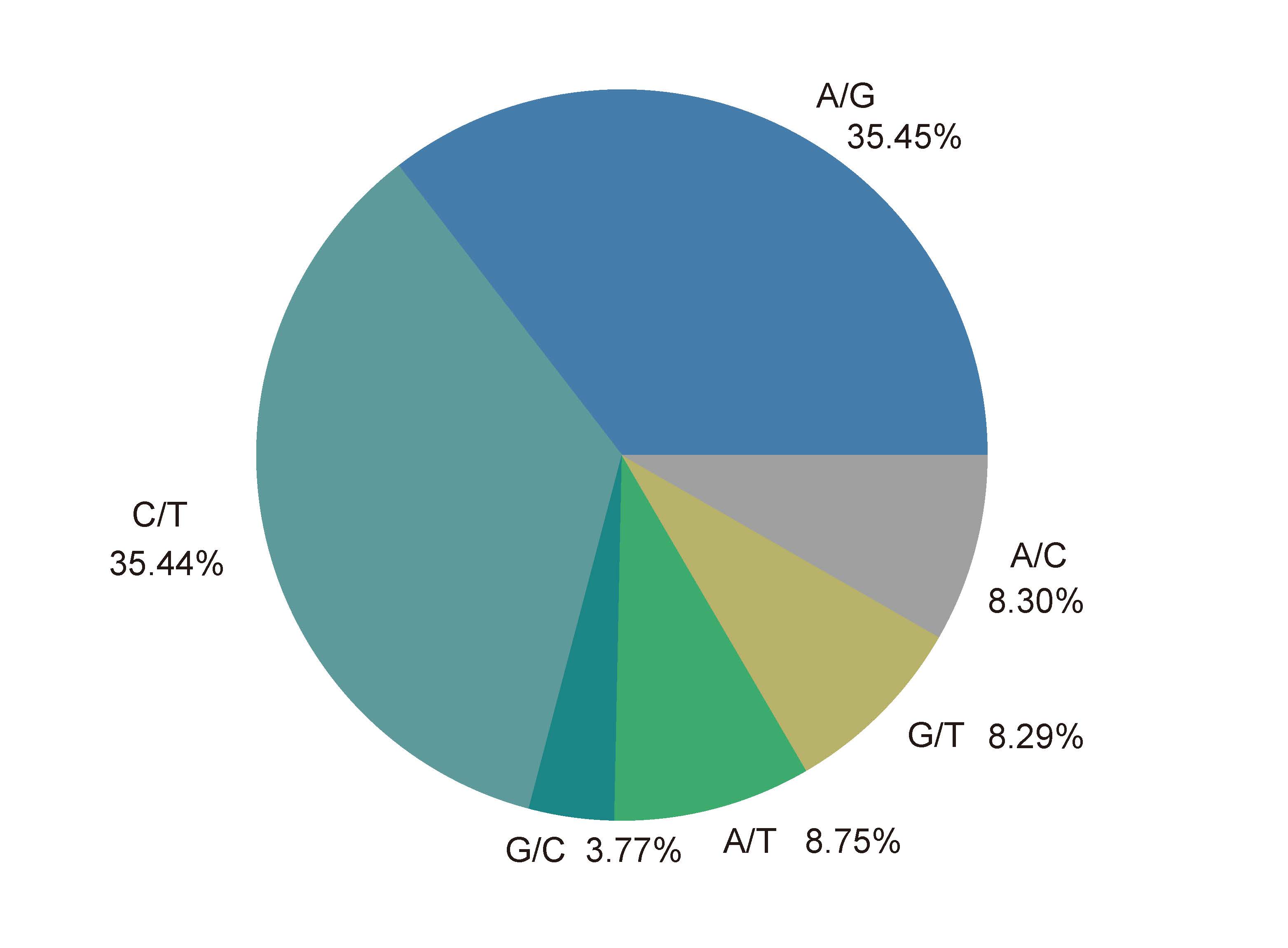

Supplement: Supplementary file 1 [file ijms-24-12662-s001.zip › Figure S3. Alternative splicing sites.jpg]

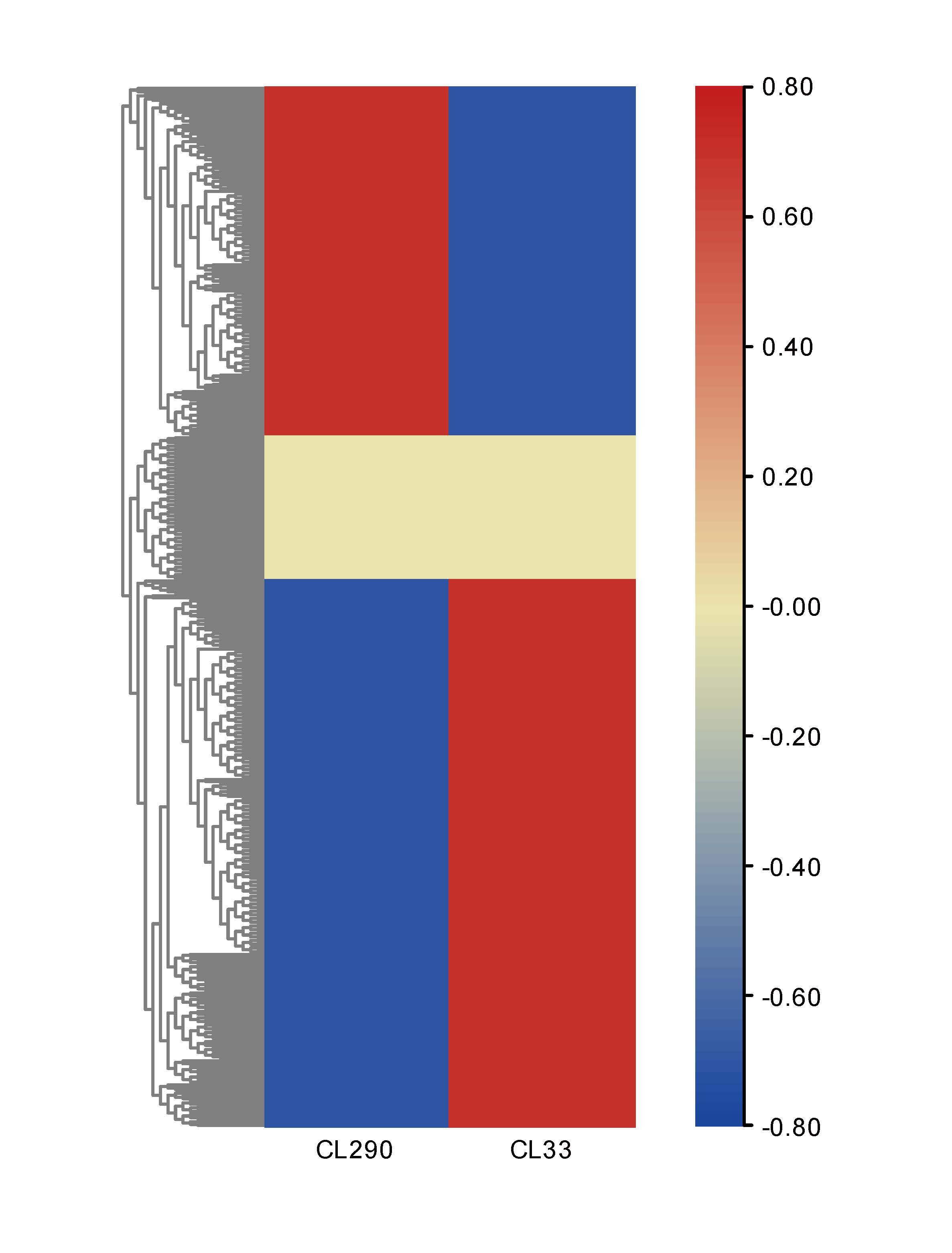

Supplement: Supplementary file 1 [file ijms-24-12662-s001.zip › Figure S4. Expression of CL290 and CL33.jpg]
